# Supplementary material for: Alteration of Lipid Metabolism in Patients with IPF and Its Association with Disease Severity and Prognosis: A Case–Control Study
Source: Int J Mol Sci. 2025 Jun 17;26(12):5790. doi: 10.3390/ijms26125790 (PMC12192574; doi:10.3390/ijms26125790)
Supplement: Supplementary file 1 [file ijms-26-05790-s001.zip › ijms-3664953-supplementary.pdf]

Supplementary Material

Supplementary Table S1: Marker of patients with IPF, stratified by GAP index (I vs II and III), and controls (median and interquartile range)

	Overall (N=100)	Controls (N=50)	Patients with Gap Index I<br>N=27 (1 missing all markers)	Patients with Gap Index II e III<br>N=21 (2 missing all markers)	p
	Median [I-III quartiles]				
**Cholesterol synthesis precursors**					
lathosterol (µg/L)	1566.56 [1344.20 - 1915.60]	1767.94 [1405.63 - 2113.48]	1626.06 [1520.54- 1846.65]	1115.80 [885.36 - 1344.20]	<0.001
desmosterol (µg/L)	886.92 [757.36 - 1066.76]	984.84 [820.04 - 1104.27]	985.20 [860.18 - 1140.33]	700.48 [516.96 - 779.96]	<0.001
lanosterol (µg/L)	266.72 [200.64 - 336.96]	315.67 [245.73 - 358.85]	259.52 [218.77 - 312.84]	177.36 [153.12 - 192.76]	<0.001
**Oxidative stress markers**					
7bOHC (µg/L)	19.27 [16.18 - 28.18]	16.31 [12.27 - 18.42]	22.74 [20.60 - 25.57]	40.39 [33.23 - 52.71]	<0.001
7aOHC (µg/L)	15.39 [12.42 - 24.17]	12.47 [9.61 - 14.92]	18.87 [15.21 - 21.57]	35.33 [31.41 - 39.29]	<0.001
7KC *(µg/L)	33.53 [24.16 - 44.75]	24.87 [16.88 - 32.81]	37.06 [33.21 - 40.24]	67.87 [58.13 - 73.65]	<0.001
5,6a-epoxy (µg/L)	57.21 [50.05 - 67.81]	51.55 [35.88 - 56.94]	58.96 [54.34 - 66.73]	81.09 [75.56 - 84.84]	<0.001
5,6b-epoxy (µg/L)	53.81 [47.81 - 62.56]	50.05 [42.49 - 55.00]	56.19 [50.94 - 61.04]	75.65 [61.50 - 82.85]	<0.001
triol (µg/L)	18.62 [15.31 - 27.66]	15.48 [12.23 - 17.43]	23.12 [19.47 - 26.73]	35.61 [30.94 - 40.97]	<0.001
24OHC (µg/L)	66.12 [58.00 - 73.28]	69.84 [59.76 - 78.08]	67.26 [61.40 - 74.17]	56.92 [50.12 - 58.40]	<0.001
**Mitochondrial oxysterol - marker of mitochondrial function**					

27OHC (µg/L)	116.88 [102.00 - 134.04]	129.32 [116.70 - 158.23]	114.50 [103.28 - 127.42]	92.68 [85.96 - 99.16]	<0.001
**Inflammation biomarkers**					
Arachidonic acid (µg/L)	122085.00 [107250.00 - 140715.00]	135472.50 [125925.00 - 153588.75]	111412.50 [103998.75 - 125865.00]	89175.00 [74010.00 - 105225.00]	<0.001
DHA (µg/L)	125.64 [96.27 - 142.23]	135.30 [122.57 - 153.09]	126.27 [110.91 - 135.13]	78.75 [73.65 - 88.74]	<0.001
**Very long-chain fatty acids**					
C22:1 brassidic (µg/L)	124.36 [102.20 - 161.36]	149.40 [124.24 - 175.62]	118.22 [101.86 - 142.11]	78.56 [68.48 - 95.16]	<0.001
C22:1 erucic (µg/L)	1289.08 [1088.44 - 1445.56]	1293.06 [1165.99 - 1743.96]	1352.26 [1068.23 - 1428.55]	1049.36 [894.12 - 1299.60]	0.001
C22 (µg/L)	2638.60 [2273.60 - 3065.20]	2859.52 [2488.48 - 3214.95]	2777.28 [2473.71 - 3145.14]	2227.32 [1775.16 - 2336.96]	<0.001
C24:1 (µg/L)	2041.28 [1398.84 - 2481.12]	1454.65 [1262.82 - 2029.44]	2054.10 [1944.03 - 2335.15]	2740.00 [2427.16 - 3009.48]	<0.001
C24 (µg/L)	814.45 [645.12 - 926.35]	679.82 [533.67 - 814.16]	833.12 [706.96 - 921.38]	1009.12 [897.27 - 1120.28]	<0.001
C26:1 (µg/L)	86.60 [76.10 - 98.03]	80.50 [71.16 - 91.49]	91.78 [78.26 - 100.52]	102.58 [93.30 - 115.56]	<0.001
C26 (µg/L)	223.93 [192.19 - 250.94]	193.64 [167.92 - 219.34]	229.85 [217.36 - 242.35]	271.20 [255.65 - 301.21]	<0.001

Supplementary Table S2. Multivariable logistic models results excluding persons on lipid-lowering therapies (statins or ezetimibe) comparing IPF patients versus controls,(N = 60) and less severe (GAP stage I) vs more severe patients (GAP stage II and III) (n=29), adjusted for age and sex. As markers have been centered and scaled, OR represents the change in the response variable for a standard deviation change in the marker.

	IPF pts vs controls			GAP II,III vs I		
	**OR[1]**	**95% CI[1]**	**p-value**	**OR[1]**	**95% CI[1]**	**p-value**
**lathosterol**	0.487	0.231, 0.905	0.037	0.088	0.007, 0.398	0.015
**desmosterol**	0.769	0.439, 1.315	0.3	0.074^	0.010, 0.530	0.010
**lanosterol**	0.415	0.208, 0.754	0.007	0.126	0.013, 0.496	0.018
**27OHC**	0.093	0.029, 0.228	<0.001	0.055^	0.006, 0.510	0.011
**Arachidonic acid**	0.075	0.023, 0.187	<0.001	0.041	0.001, 0.308	0.025
**DHA**	0.166^	0.064, 0.431	<0.001	0.054^	0.005, 0.582	0.016
**C22:1 brassidic**	0.171	0.052, 0.407	<0.001	0.023	0.000, 0.255	0.033
**C22:1 erucic**	0.428	0.210, 0.785	0.011	0.429	0.137, 1.174	0.11
**C22**	0.53	0.276, 0.938	0.039	0.108	0.011, 0.429	0.011
**C24:1**	3.365	1.732, 7.612	0.001	19.679^	1.876, 206.481	0.013
**C24**	3.334	1.695, 7.777	0.002	11.249^	1.535, 82.420	0.017
**C26:1 `**	3.877	1.899, 9.499	<0.001	8.330	2.005, 79.725	0.018
**C26**	8.637	3.119, 35.505	<0.001	42.273^	0.911, 1,962.393	0.056

[1] OR = Odds Ratio for a standard deviation increase in the marker, CI = Confidence Interval;

^mixed bias-reducing adjusted score equations (https://doi.org/10.1002/wics.1296)
